# Supplementary figures and images for: A toolset of constitutive promoters for metabolic engineering of Rhodosporidium toruloides
Source: Microb Cell Fact. 2019 Jun 29;18:117. doi: 10.1186/s12934-019-1167-0 (PMC6599526; doi:10.1186/s12934-019-1167-0)

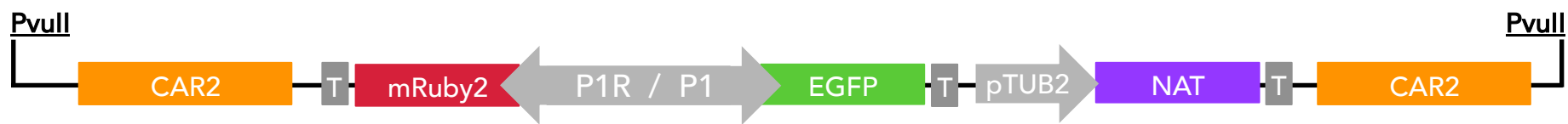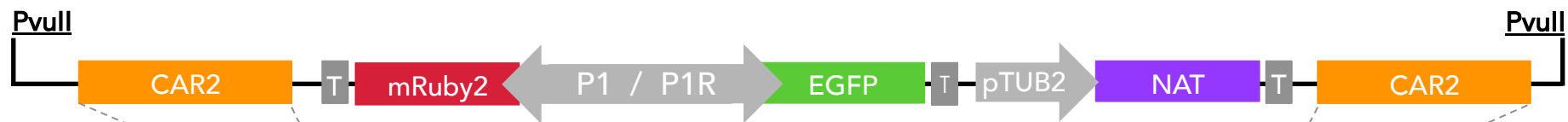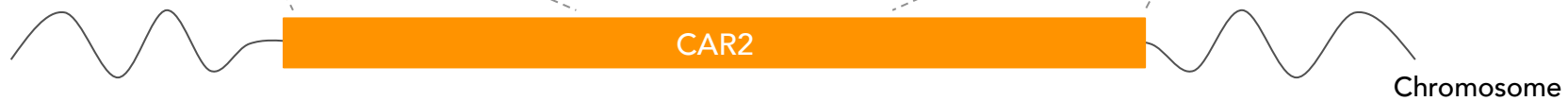

Supplement: Supplementary file 1 — Additional file 1: Figure S1. Schematic representation of the expression cassettes to be inserted into the R. toruloides genome through homologous recombination. Representative design of the constructs in orientation 1 (top), showing promoter P1 driving EGFP expression and P1R driving mRuby2 and orientation 2 (lower), where the promoter fragment has been reversed. Promoter-reporter constructs, paired with a NAT cassette (conferring resistance to nourseothricin), are flanked by CAR2 gene fragments to facilitate integration of the cassette into the CAR2 locus of the R. toruloides chromosome. [file 12934_2019_1167_MOESM1_ESM.pdf]

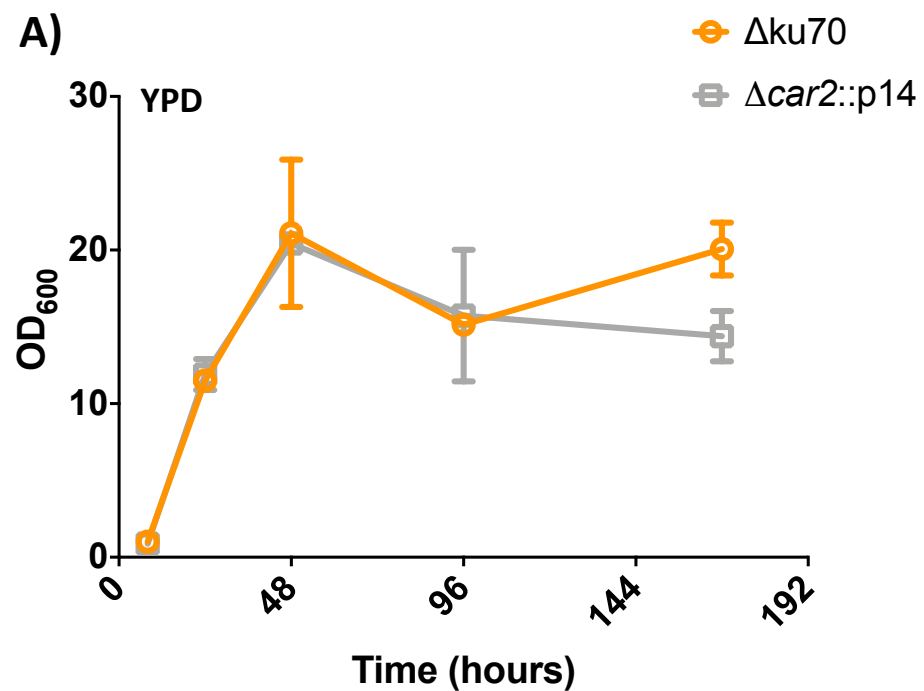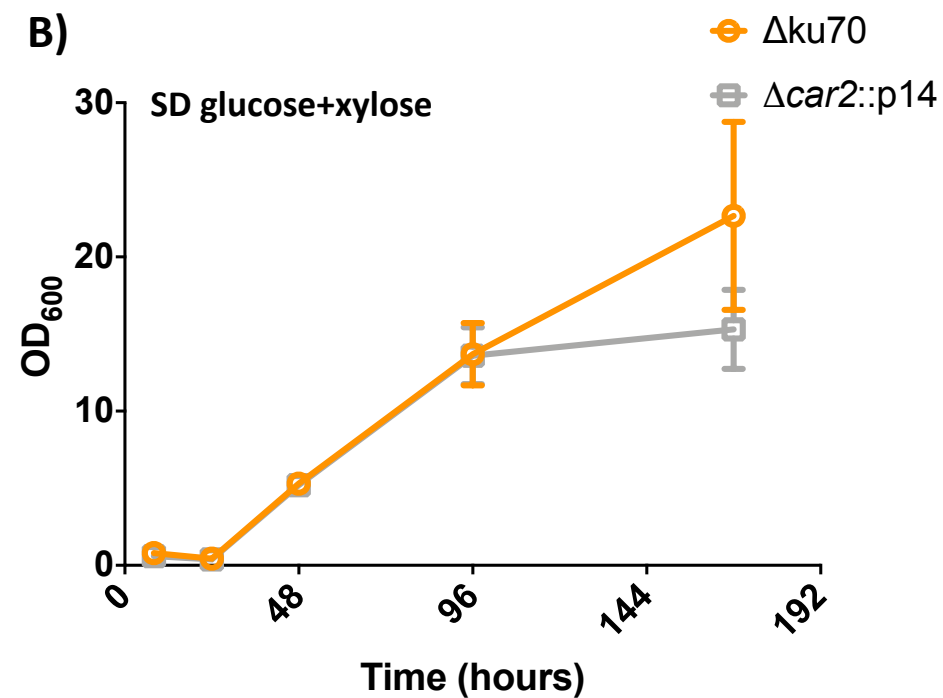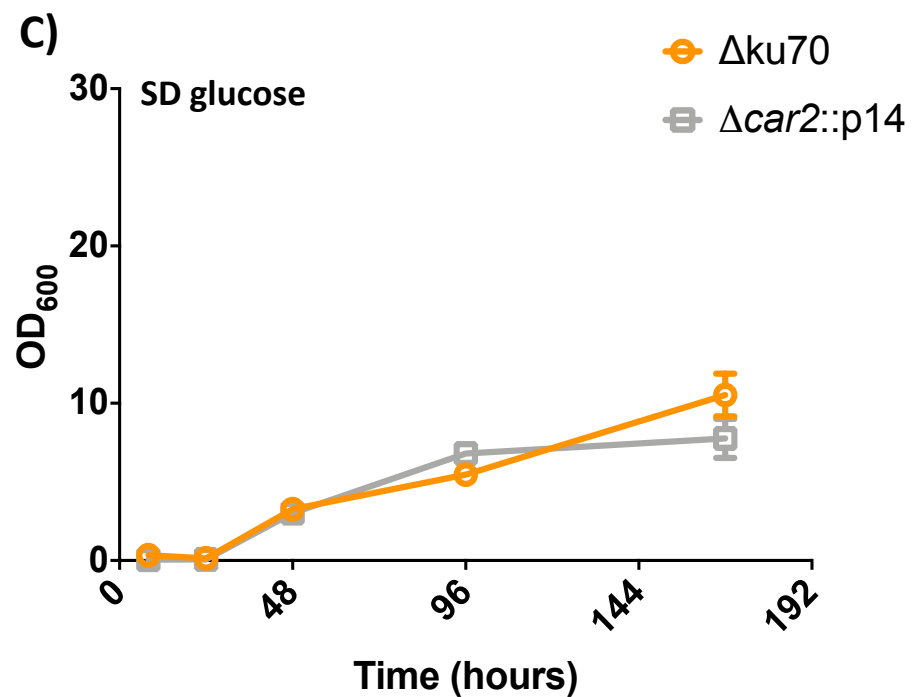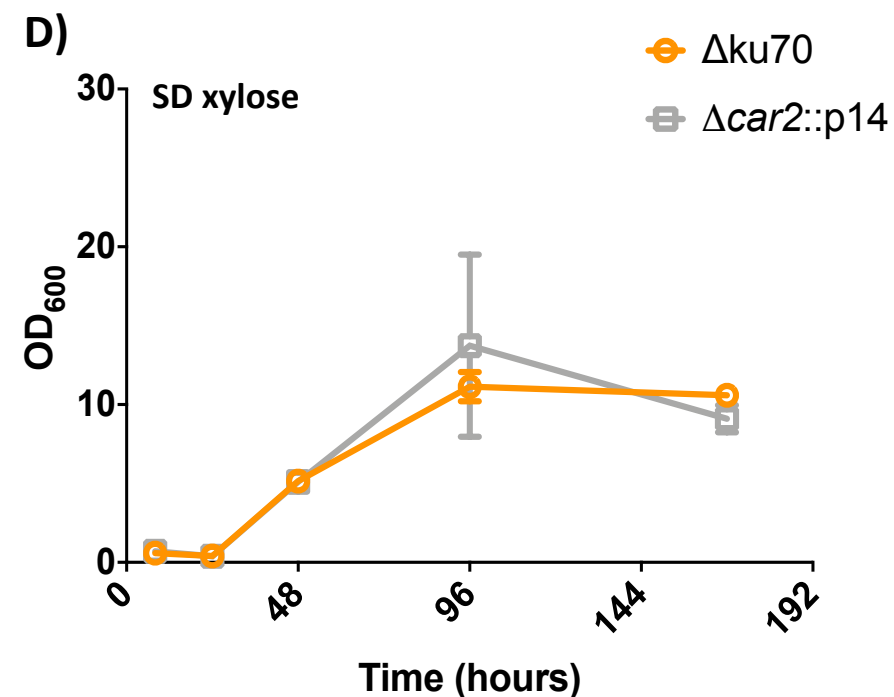

Supplement: Supplementary file 2 — Additional file 2: Figure S2. Growth of the parental R. toruloides ∆ku70 strain and a strain harboring promoter construct P14. Growth was monitored by OD600 measurements, taken at 8, 24, 48, 96 and 168 h from strains grown in: YPD (A); SD supplemented with 1% (w/v) each of glucose and xylose (B); SD supplemented with 1% (w/v) glucose (C); and SD supplemented with 1% (w/v) xylose (D). [file 12934_2019_1167_MOESM2_ESM.pdf]

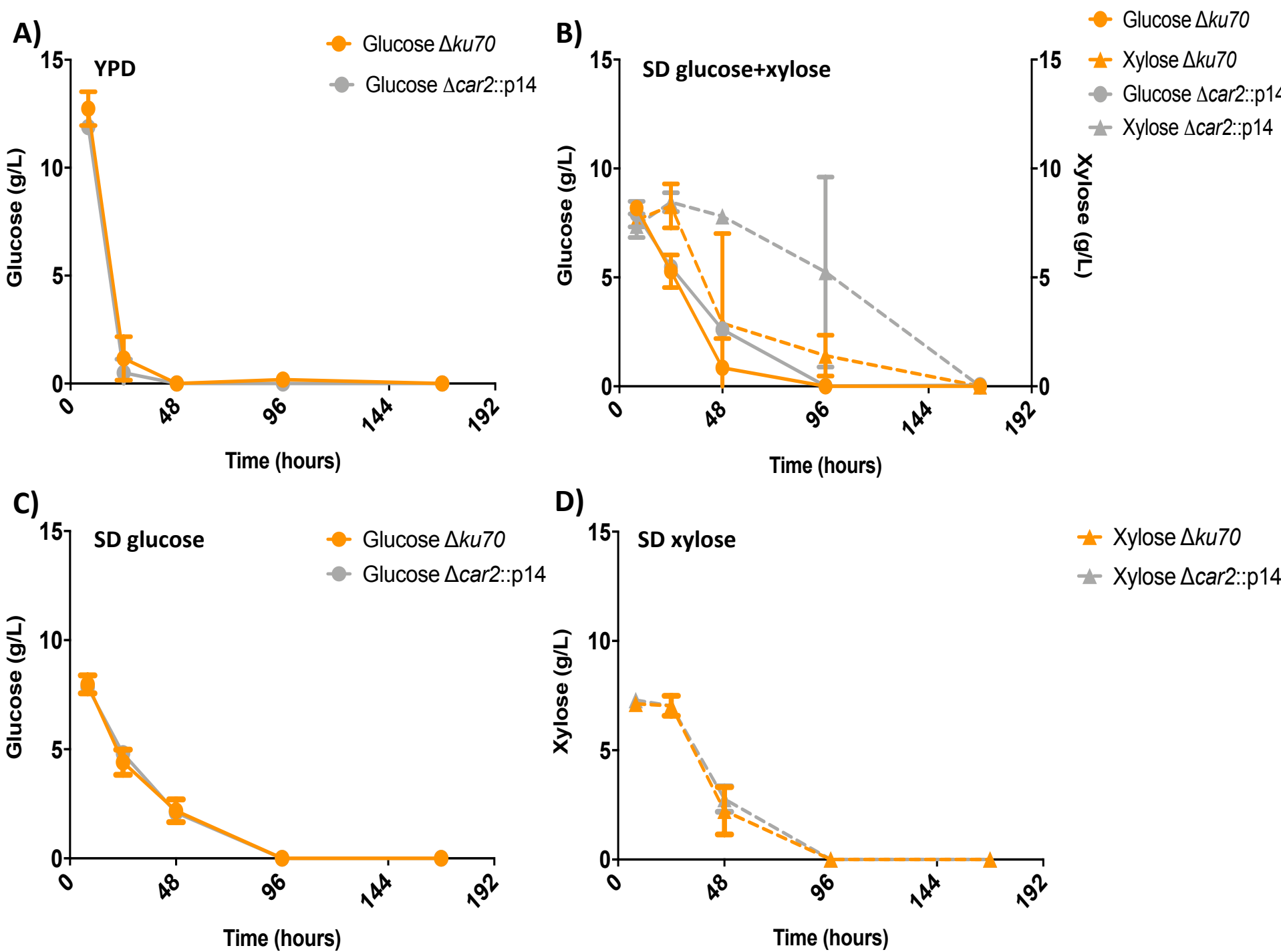

Supplement: Supplementary file 3 — Additional file 3: Figure S3. Glucose and xylose consumption in the four media used for this study. Two strains are shown: the parental R. toruloides ∆ku70 strain and strain harboring promoter construct P14. Sugars were quantified by HPLC from samples taken at 8, 24, 48, 96 and 168 h from strains grown in: YPD (A); SD supplemented with 1% (w/v) each of glucose and xylose (B); SD supplemented with 1% (w/v) glucose (C); and SD supplemented with 1% (w/v) xylose (D). [file 12934_2019_1167_MOESM3_ESM.pdf]

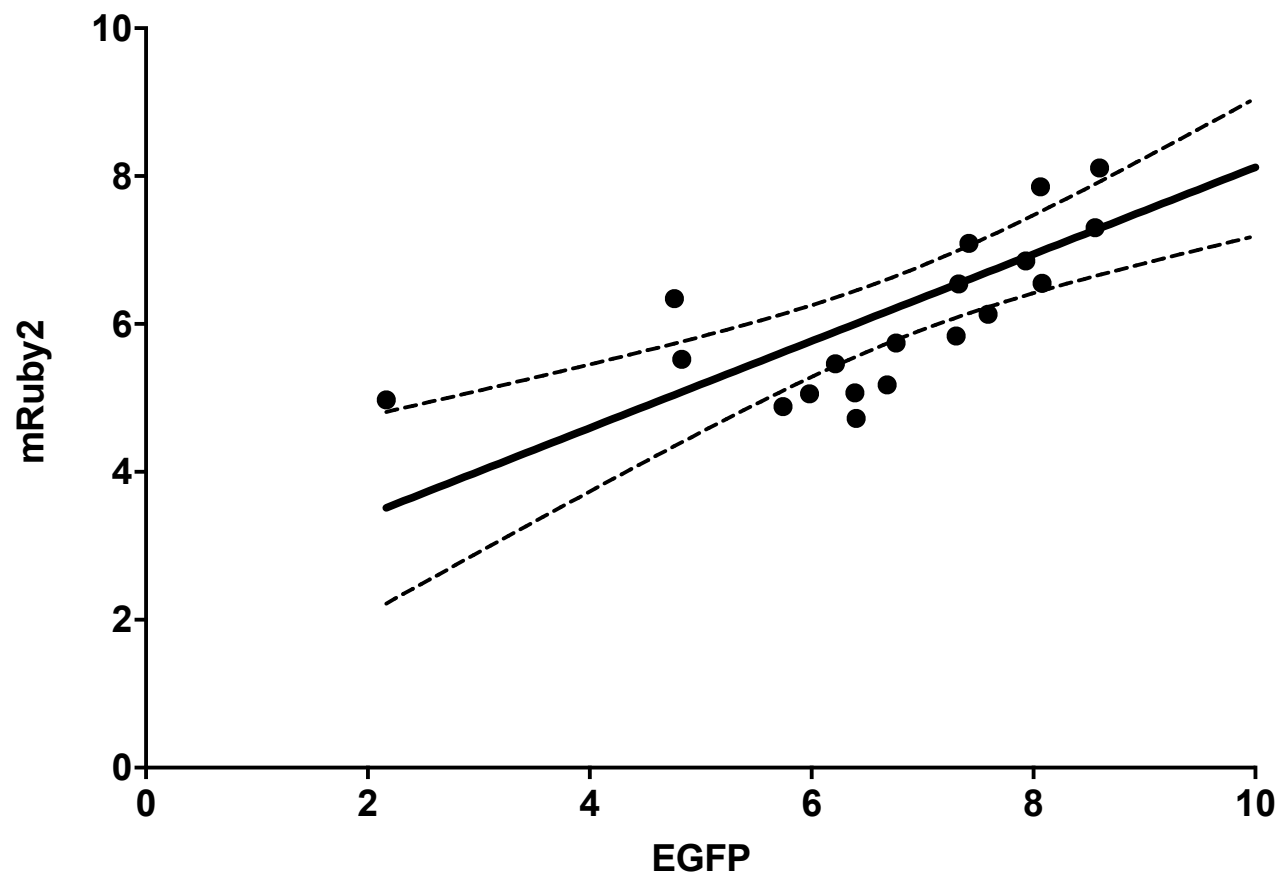

Supplement: Supplementary file 4 — Additional file 4: Figure S4. Correlation between EGFP and mRuby2 expression from promoter P9 in all 4 media and all 5 time points. Fluorescence expression values are on a Log2 scale. R2 = 0.5537 for a P value of 0.0002. Solid line is the linear regression and dashed lines are 95% confidence interval. [file 12934_2019_1167_MOESM4_ESM.pdf]
