# Supplementary material for: A toolset of constitutive promoters for metabolic engineering of Rhodosporidium toruloides
Source: Microb Cell Fact. 2019 Jun 29;18:117. doi: 10.1186/s12934-019-1167-0 (PMC6599526; doi:10.1186/s12934-019-1167-0)
Supplement: Supplementary file 5 — Additional file 5: Table S1. Promoters investigated and strains generated in this study. [file 12934_2019_1167_MOESM5_ESM.docx]

**Table S1. Promoters investigated and strains generated in this study.**

Promoters selected from RNA-sequencing data are listed with their locations in the *R. toruloides* genome, links to their respective constructs in the Joint BioEnergy Institute (JBEI) registry, and annotations of putative gene functions. Promoter pairs that span complete intergenic regions and are therefore predicted to be bidirectional are represented by two listings, annotated as P1 and P1R, etc. Protein ID and Transcript ID refers to the *Rhodosporidium toruloides* IFO0880 v4.0 genome sequence, which can be found on the Joint Genome Institute (JGI) MycoCosm site: https://genome.jgi.doe.gov/Rhoto_IFO0880_4/Rhoto_IFO0880_4.home.html.

|  | **JBEI Registry Plasmid ID**  **(Orientation 1)** | **JBEI Registry Plasmid ID (Orientation 2)** | **Protein ID** | **Transcript ID** | **Promoter Size**  **(bp)** | **Annotation** |
| --- | --- | --- | --- | --- | --- | --- |
| **P1** | **JPUB_013255** | **JPUB_013313** | **10325** | **10453** | **366** | **K02984: RP-S3Ae, RPS3A; small subunit ribosomal protein S3Ae** |
|  |  |  | **10324** | **10452** | **366** | **BLAST: 40S ribosomal protein S1 [Exophiala sideris]** |
| **P2** | **JPUB_013257** | **JPUB_013315** | **15909** | **16037** | **218** | **K04564: SOD2; superoxide dismutase, Fe-Mn family** |
|  |  |  | **15908** | **16036** | **218** | **K01012: bioB; biotin synthase** |
| **P3** | **JPUB_013259** | **JPUB_013317** | **11021** | **11149** | **997** | **K15040: VDAC2; voltage-dependent anion channel protein 2** |
| **P4** | **JPUB_013261** | **JPUB_013319** | **14514** | **14642** | **997** | **K02925: RP-L3e, RPL3; large subunit ribosomal protein L3e** |
| **P5** | **JPUB_013263** | **JPUB_013321** | **10055** | **10183** | **997** | **K02969: RP-S20e, RPS20; small subunit ribosomal protein S20e** |
| **P6** | **JPUB_013265** | **JPUB_013323** | **15265** | **15393** | **997** | **Domain of Unknown Function** |
| **P7** | **JPUB_013267** | **JPUB_013325** | **10613** | **10741** | **943** | **K00134: GAPDH, gapA; glyceraldehyde 3-phosphate dehydrogenase** |
| **P8** | **JPUB_013269** | **JPUB_013327** | **8752** | **8880** | **601** | **KOG0004: Ubiquitin/40S ribosomal protein S27a fusion** |
|  |  |  | **8751** | **8879** | **601** | **K02922: RP-L37e, RPL37; large subunit ribosomal protein L37e** |
| **P9** | **JPUB_013271** | **JPUB_013329** | **9231** | **9359** | **453^*^** | **K11254: H4; histone H4** |
|  |  |  | **9232** | **9360** | **453^*^** | **K11253: H3; histone H3** |
| **P10** | **JPUB_013273** | **JPUB_013331** | **13099** | **13227** | **755** | **K08770: UBC; ubiquitin C** |
| **P11** | **JPUB_013275** | **JPUB_013333** | **14295** | **14423** | **124** | **K02979: RP-S28e, RPS28; small subunit ribosomal protein S28e** |
|  |  |  | **14294** | **14422** | **124** | **K02989: RP-S5e, RPS5; small subunit ribosomal protein S5e** |
| **P12** | **JPUB_013277** | **JPUB_013335** | **14295** | **14423** | **986** | **K02979: RP-S28e, RPS28; small subunit ribosomal protein S28e** |
|  |  |  | **14294** | **14422** | **986** | **K02989: RP-S5e, RPS5; small subunit ribosomal protein S5e** |
| **P13^**^** | **JPUB_013337** | **JPUB_013279** | **9006** | **9134** | **794** | **K02947: RP-S10e, RPS10; small subunit ribosomal protein S10e** |
|  |  |  | **9007** | **9135** | **794** | **K18027: PTPN3, PTPH1; tyrosine-protein phosphatase non-receptor type 3** |
| **P14** | **JPUB_013281** | **JPUB_013339** | **12693** | **12821** | **997** | **K03231: EEF1A; elongation factor 1-alpha** |
| **P15** | **JPUB_013283** | **JPUB_013341** | **12704** | **12832** | **997** | **K05863: SLC25A4S, ANT; solute carrier family 25 (mitochondrial adenine nucleotide translocator), member 4/5/6/31** |
| **P16** | **JPUB_013285** | **JPUB_013343** | **14937** | **15065** | **997** | **K02978: RP-S27e, RPS27; small subunit ribosomal protein S27e** |
| **P17** | **JPUB_013287** | **JPUB_013345** | **15825** | **15953** | **1001** | **Hypothetical protein** |
| **P18** | **JPUB_013289** | **JPUB_013347** | **11331** | **11459** | **1189** | **K01647: CS, gltA; citrate synthase** |
| **P19** | **JPUB_013291** | **JPUB_013349** | **16419** | **16547** | **941** | **K02958: RP-S15e, RPS15; small subunit ribosomal protein S15e** |
|  |  |  | **16418** | **16546** | **941** | **K02943: RP-LP2, RPLP2; large subunit ribosomal protein LP2** |
| **P20** | **JPUB_013293** | **JPUB_013351** | **8813** | **8941** | **856** | **K03768: PPIB, ppiB; peptidyl-prolyl cis-trans isomerase B (cyclophilin B)** |
| **P21** | **JPUB_013295** | **JPUB_013353** | **9048** | **9176** | **335** | **K03094: SKP1, CBF3D; S-phase kinase-associated protein 1** |
|  |  |  | **9047** | **9175** | **335** | **Hypothetical protein** |
| **P22** | **JPUB_013297** | **JPUB_013355** | **12216** | **12344** | **513** | **KOG1817: Ribonuclease** |
|  |  |  | **12215** | **12343** | **513** | **Hypothetical protein** |
| **P23** | **JPUB_013299** | **JPUB_013357** | **12844** | **12972** | **570** | **HMMPfam:Domain of unknown function (DUF543):PF04418** |
| **P24** | **JPUB_013301** | **JPUB_013359** | **13007** | **13135** | **130** | **KOG3469: Cytochrome c oxidase, subunit VIa/COX13** |
|  |  |  | **13006** | **13134** | **130** | **HMMPfam:GDSL/SGNH-like Acyl-Esterase family found in Pmr5 and Cas1p:PF13839,SUPERFAMILY::SSF52266** |
| **P25** | **JPUB_013303** | **JPUB_013361** | **13608** | **13736** | **373** | **HMMPfam:Ubiquinol-cytochrome-c reductase complex subunit (QCR10):PF09796** |
|  |  |  | **13607** | **13735** | **373** | **K10859: ALKBH2; alpha-ketoglutarate-dependent dioxygenase alkB homolog 2** |
| **P26** | **JPUB_013305** | **JPUB_013363** | **13614** | **13742** | **222** | **K00411: UQCRFS1, RIP1, petA; ubiquinol-cytochrome c reductase iron-sulfur subunit** |
| **P27** | **JPUB_013307** | **JPUB_013365** | **14603** | **14731** | **643** | **K17279: REEP5_6; receptor expression-enhancing protein 5/6** |
| **P28** | **JPUB_013309** | **JPUB_013367** | **15467** | **15595** | **382** | **K12845: SNU13, NHP2L; U4/U6 small nuclear ribonucleoprotein SNU13** |
|  |  |  | **15466** | **15594** | **382** | **KOG2633: Hismacro and SEC14 domain-containing proteins** |
| **P29** | **JPUB_013311** | **JPUB_013369** | **15484** | **15612** | **763** | **K04393: CDC42; cell division control protein 42** |
|  |  |  | **15485** | **15613** | **763** | **K12868: SYF2; pre-mRNA-splicing factor SYF2** |

^*^ The sequence for Promoter 9 contains an intron inside the start codon and the promoter length in this case includes this intron and start codon (ATG).

^**^Plasmids for Promoter 13 are named forward in orientation 2 and reverse in orientation 1 because they were built in opposite directions.
